# Silicone Embolism Syndrome Causing Altered Mental Status and Respiratory Failure After an Unlicensed Gluteal Silicone Injection: A Case Report

**DOI:** 10.5811/cpcem.1441

**Published:** 2024-03-26

**Authors:** Christopher Lin, Jeremiah Robison

**Affiliations:** Saint Barnabas Hospital, Department of Emergency Medicine, Bronx, New York

**Keywords:** *case report*, *silicone embolization syndrome*, *ARDS*, *cosmetic surgery*

## Abstract

**Introduction:**

Unlicensed cosmetic procedures, which come at increased risk of infection and potential surgical complications, have introduced new challenges in healthcare. Physicians should be aware of presentations that may arise secondary to these procedures.

**Case Report:**

We describe a case in which a previously healthy, 28-year-old female presented with new-onset seizures and acute respiratory distress syndrome (ARDS) in the setting of a recent cosmetic procedure with silicone injections to the gluteal region. The patient’s hospital course was complicated by altered mental status, respiratory failure, rapid hemodynamic compromise, and eventual death.

**Conclusion:**

In rare cases, one possible complication of cosmetic procedures is silicone embolism syndrome, which is characterized by pneumonitis, alveolar hemorrhage, and ARDS. The patient described in this report also experienced neurologic symptoms including seizure and altered mental status. This is a clinical diagnosis that relies upon thorough history-taking and detailed physical exam. Documentation on this phenomenon is limited, and medical management has not yet been standardized for this condition. Morbidity and mortality remain high.

## INTRODUCTION

Unlicensed cosmetic procedures have resulted in complications including infection and cosmetic deformities. Treatment of these complications may require antibiotic therapy, hospital admission, and at times surgical intervention.^1^^–^^3^ Limited documentation in cases of unlicensed cosmetic surgery brings additional challenges to uncovering history and patient management.^4^^–^^7^ Silicone injections are not approved by the US Food and Drug Administration due to their association with infection, permanent disfigurement, embolism, and death. These restrictions have prompted individuals to seek certain cosmetic procedures by unlicensed practitioners within the United States or abroad. In these cases, practitioners may be unqualified for the cosmetic interventions performed. Additionally, the cosmetic fillers used may be prone to impurities due to the absence of regulations.^2^

The increased prevalence of medical tourism for cosmetic procedures has contributed to complications secondary to unlicensed medical practice. Within the US, the majority of patients who undergo medical tourism are Hispanic females; travel destinations are primarily within Latin America, with the Dominican Republic the most frequented destination.^1^ While data is limited, it is suspected that individuals pursue cosmetic options in foreign countries due to lower financial costs; one study found that the majority of patients affected are Medicaid recipients and of lower socioeconomic backgrounds.^2^

Liquid silicone (polydimethylsiloxane) is a commonly used substance for cosmetic injections, primarily as a soft tissue expander. In rare cases, silicone embolism syndrome (SES), a complication associated with illicit cosmetic modifications involving silicone injections, can occur. This condition is associated with pneumonitis, alveolar hemorrhage, and acute respiratory distress syndrome (ARDS).^4^^–^^7^ Current literature suggests that the pulmonary findings are caused by a silicone embolism from direct intravenous injection. Alternatively, they may be caused after local tissue destruction. After reaching lung tissue, the silicone triggers a cascade of inflammatory responses including proteolysis and recruitment of neutrophils.^4^ The embolus itself can result in occlusion of microvasculature, further affecting pulmonary function. Pulmonary hemorrhage and edema can occur rapidly.

Symptoms have been reported as early as within 72 hours of cosmetic surgery. However, delayed presentations up to 18 years after initial surgery have been documented.^4^^,^^8^ Due to the limited number of case reports regarding this condition, the average time course from surgery to symptom onset is unclear. Presentations vary, although most reports indicate symptom onset within hours to days. Imaging reports are largely consistent between cases, with computed tomography (CT) revealing diffuse alveolar infiltrates or ground-glass opacities.^4^^–^^8^

## CASE REPORT

An otherwise healthy, 28-year-old Hispanic female with no known past medical history was brought to the emergency department by emergency medical services. According to the history provided, the patient had been walking with a friend before experiencing sudden onset abdominal pain followed by seizure activity. After an episode of emesis and repeat seizure during initial triage evaluation, the patient was taken to the resuscitation bay for further evaluation. Further history-taking from family revealed that the patient had attended a “cosmetic party” earlier that day (within the prior 24 hours) and received gluteal soft-tissue injections.

Initial vitals were notable for tachycardia with a heart rate of 116 beats per minute (bpm), and tachypnea with a respiratory rate of 34 breaths per minute. The patient was also found to be hypoxic at 70% saturation on pulse oximetry. Temperature and blood pressure were 98.3° Fahrenheit and 137/82 millimeters of mercury (mm Hg), respectively. Seizure activity was controlled with 4 milligrams (mg) of lorazepam. The patient remained unable to provide history or follow commands, with continued hypoxia and signs of respiratory distress despite supplemental oxygen with a nonrebreather mask. Due to respiratory failure, rapid sequence intubation was performed. The physical exam was notable for findings suggestive of recent gluteal injections, with surgical pen markings, injection sites, and oozing of clear liquid. After intubation, bloody fluid from the endotracheal tube was suggestive of alveolar hemorrhage.

**Image 1.**
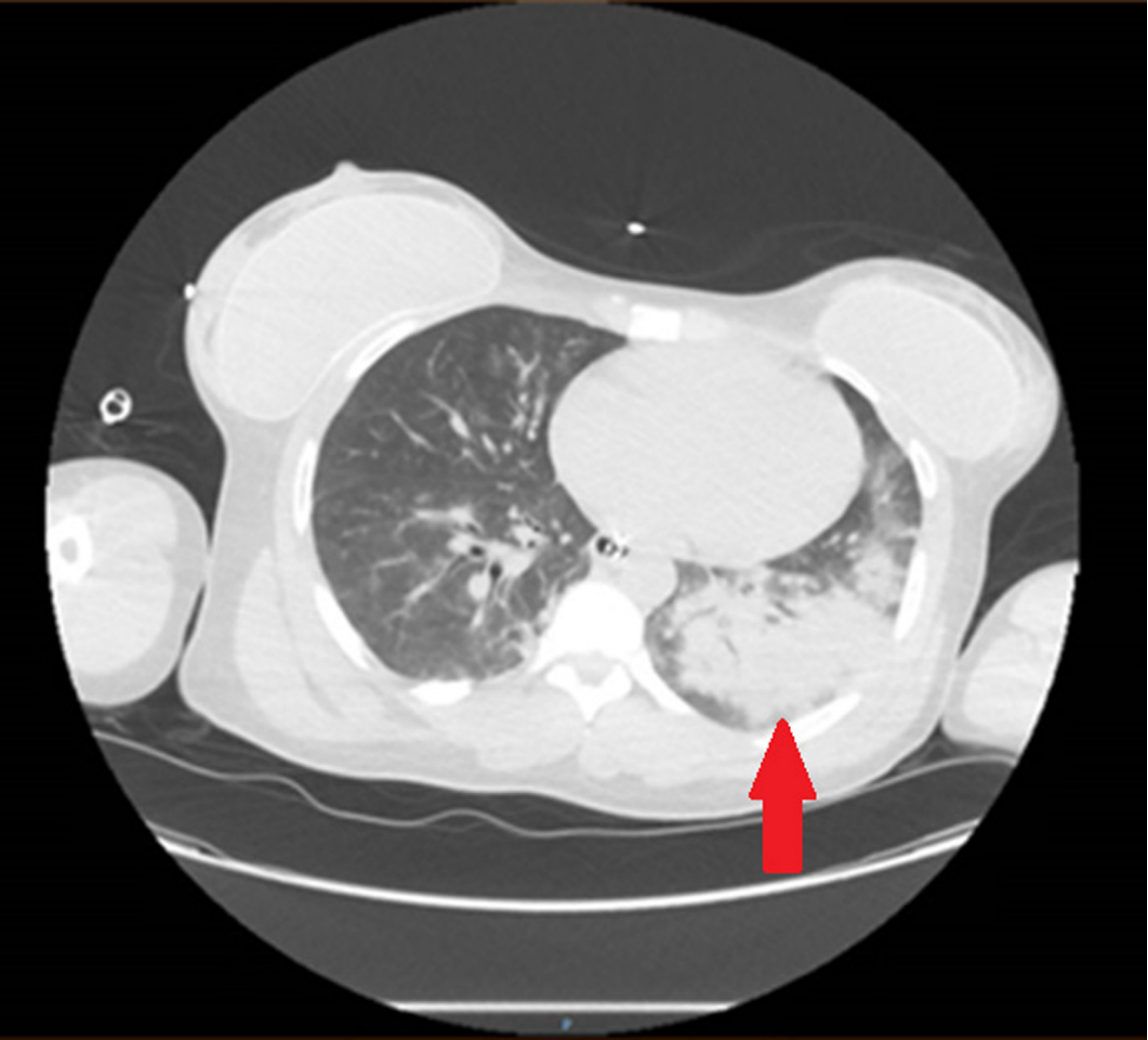
Computed tomography of the chest with contrast demonstrating bilateral pulmonary airspace disease, more pronounced in the left lung (arrow).

**Image 2.**
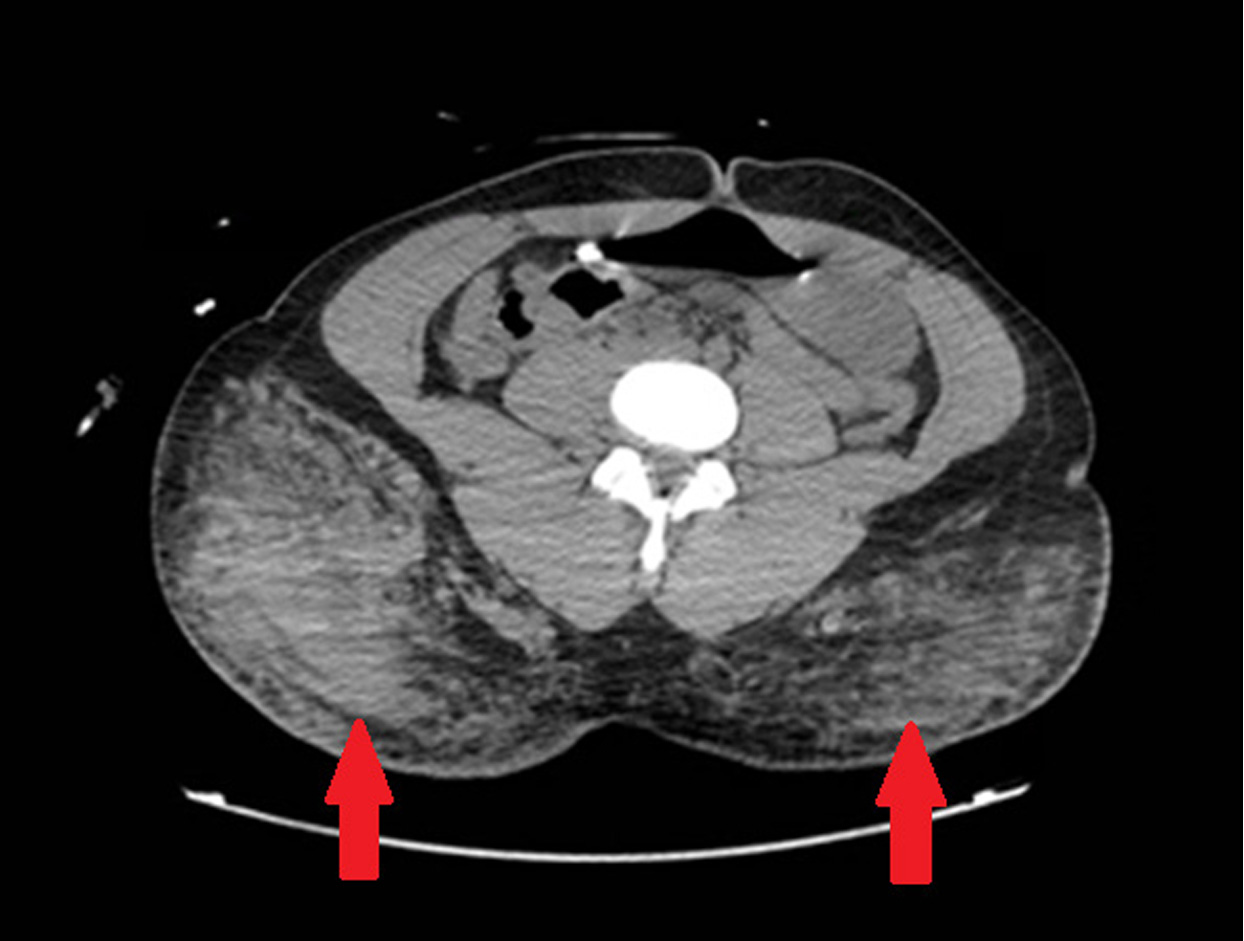
Computed tomography of the abdomen and pelvis with contrast demonstrating opacities in gluteal subcutaneous fat, suggestive of inflammation from silicone injections (arrows).

**Image 3.**
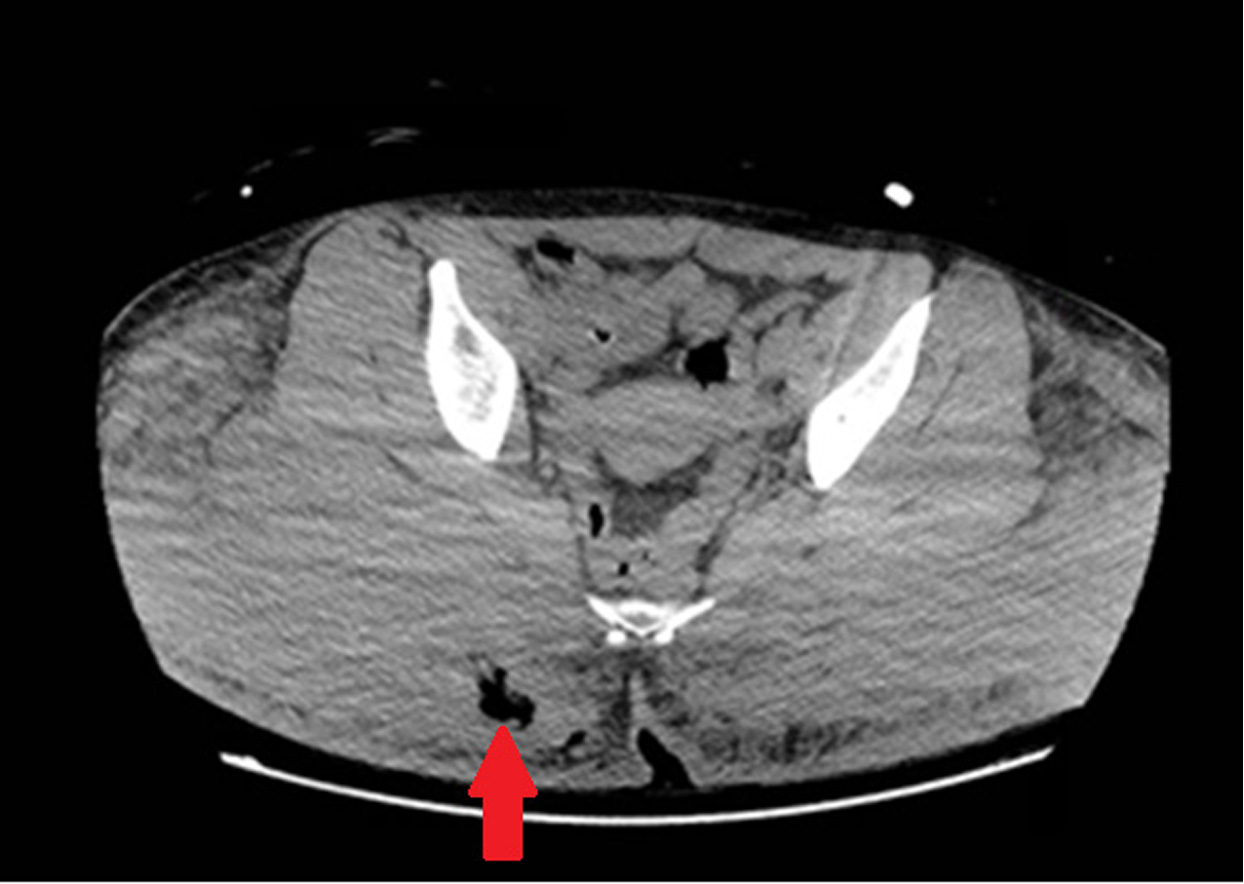
Computed tomography of the abdomen and pelvis with contrast demonstrating subcutaneous emphysema in the right gluteal region (arrow).

Chest radiograph obtained immediately after intubation demonstrated left-sided, ground-glass opacities. Computed tomography demonstrated extensive bilateral pulmonary airspace disease as well as opacities in the gluteal area suggestive of cosmetic injections, consistent with the physical exam (Images 1 and 2). A subsegmental pulmonary embolism was also present. Broad spectrum antibiotics were initiated due to concern for a surgical site infection, given subcutaneous emphysema also present on CT (Image 3). Blood gas results were notable for a lactic acidosis with a pH of 7.24 (reference range 7.35–7.45). A decreased ratio of arterial oxygen partial pressure to fractional inspired oxygen of 73 was also present, suggestive of severe ARDS. Altered mental status and respiratory failure are suspected to be secondary to effects of silicone emboli.

The patient was admitted to the intensive care unit for further management. The hospital course was complicated by further hemodynamic instability and worsening acidemia. The patient experienced rapidly worsening tachycardia and hypotension, with the heart rate increasing to 144 bpm and mean arterial pressure decreasing to 60 mm Hg within a one-hour time span. Increasing troponin levels from 0.49 nanograms per milliliter (ng/mL) to 1.64 ng/mL (reference range 0–0.5 ng/mL) were also noted. Seven hours after arrival at the hospital, the patient experienced a cardiac arrest. Despite aggressive intervention involving one hour of cardiopulmonary resuscitation and the administration of alteplase for suspected embolism, the patient was pronounced dead.

Review by the medical examiner later confirmed initial clinical impressions. Exam was remarkable for multiple puncture marks at the gluteal region. Multiple cysts filled with viscous material and granulation tissue were present. The patient was noted to have multiple emboli systemically, present in the lungs, kidneys, heart, and brain. Alveolar hemorrhage of the lungs was noted; histological analysis demonstrated lipoid material in the vasculature as well as within macrophages. Microscopic analysis of embolic material was consistent with silicone. The autopsy report identified evidence of silicone embolism to microvascular structures of the brain, with ischemic changes noted.

## DISCUSSION

Isolated case reports have documented pulmonary complications secondary to liquid silicone injections. Silicone embolism syndrome results in multiple systemic findings, including mental status changes, seizure activity, ARDS, and alveolar hemorrhage.^4^^–^^8^ Here, we describe a case of SES resulting in respiratory failure, although this was also associated with rapid hemodynamic instability and death. For this patient case, clinical findings in addition to autopsy results confirmed that recent cosmetic surgery by an unlicensed practitioner resulted in silicone embolism to multiple organ systems. Seizure activity as noted during the initial patient presentation was likely attributed to silicone embolism to the brain. These findings suggest that silicone emboli can directly affect multiple organ systems; we suspect that involvement of the central nervous system, characterized by emboli and ischemic changes within the brain, is associated with increased mortality and rapid clinical deterioration.

Although medical management of SES has not yet been standardized, of the case reports that detail this phenomenon, resolution of ARDS and overall recovery have been associated with corticosteroid use and lung-protective mechanical ventilation.^4^^–^^8^ Intravenous methylprednisolone has most commonly been used in the treatment of respiratory distress in the setting of SES. Improved outcomes are believed to be secondary to decreased airway inflammation.^4^^–^^7^ Other case studies noted improvement after the use of extracorporeal membrane oxygenation (ECMO) in the setting of respiratory failure and persistent hypotension.^9^^,^^10^ Neurologic manifestations, including altered mental status and coma, may occur and are associated with poorer outcomes.^6^^,^^9^^,^^11^ Seizure onset, as documented in this case study, is an atypical presentation that has not been extensively documented in other reports. Cases of survival have been reported; prognosis may depend on volume of silicone and pressure of injection.^4^^–^^7^

One 2019 case report describes a case of SES that responded to a regimen of methylprednisolone 125 mg every six hours. The patient in that case, who also received ECMO, recovered. Presentation was acute and within two days of a silicone injection.^11^ Another 2013 study also reported administration of methylprednisolone at the same dosing with patient survival. Onset was also within two days of a gluteal injection.^7^ These patient cases, in addition to the one described in our case report, describe a relatively quick onset of symptoms after cosmetic surgery.

With regard to clinical practice, silicone injections create a challenge in which the underlying etiology of a critically ill, otherwise healthy patient, may not be readily apparent. The location of potential cosmetic surgery may be revealed by a thorough physical exam or CT. With unlicensed procedures, a full history may not be available from health records alone. Patients may not be forthcoming with sensitive history regarding cosmetic surgery, especially if appearing not to be directly related to new-onset pulmonary changes. In critically ill patients, verbal history may not be available. Physicians should consider the diagnosis of silicone embolism in young patients with acute hemodynamic or mental status changes, when history or exam findings are suggestive of recent cosmetic surgery.

## CONCLUSION

Unlicensed cosmetic procedures have resulted in complications that introduce new medical challenges for emergency physicians. In rare cases, embolization of silicone may result in acute respiratory failure and may progress to systemic findings that include neurologic changes and cardiopulmonary compromise. Volume of silicone injection, in addition to the extent of embolization, may be factors that affect mortality. In the case we report here the sudden hemodynamic changes and mental status changes were likely secondary to widespread silicone emboli, which were identified in multiple organ systems. Early identification of potential silicone embolism syndrome and aggressive supportive care appear to be beneficial. Further studies documenting interventions and outcomes will help guide future management.
